# Healthy diet intervention reverses the progression of NASH through gut microbiota modulation

**DOI:** 10.1128/spectrum.01868-23

**Published:** 2023-11-29

**Authors:** Suraphan Panyod, Wei-Kai Wu, Meng-Yun Hu, Huai-Syuan Huang, Rou-An Chen, Yi-Hsun Chen, Ting-Chin David Shen, Chi-Tang Ho, Chun-Jen Liu, Hsiao-Li Chuang, Chi-Chang Huang, Ming-Shiang Wu, Lee-Yan Sheen

**Affiliations:** 1 Institute of Food Science and Technology, National Taiwan University, Taipei, Taiwan; 2 Center for Food and Biomolecules, National Taiwan University, Taipei, Taiwan; 3 Department of Medical Research, National Taiwan University Hospital, Taipei, Taiwan; 4 Department of Internal Medicine, College of Medicine, National Taiwan University, Taipei, Taiwan; 5 Division of Gastroenterology, University of Pennsylvania, Perelman School of Medicine, Philadelphia, Pennsylvania, USA; 6 Department of Food Science, Rutgers University, New Brunswick, New Jersey, USA; 7 Department of Internal Medicine, National Taiwan University Hospital, Taipei, Taiwan; 8 National Laboratory Animal Center, National Applied Research Laboratories, Taipei, Taiwan; 9 Graduate Institute of Sports Science, National Taiwan Sport University, Taoyuan City, Taiwan; 10 National Taiwan University, National Center for Food Safety Education and Research, Taipei, Taiwan; Huazhong University of Science and Technology, Wuhan, China

**Keywords:** non-alcoholic steatohepatitis, GAN diet, ginger essential oil, obeticholic acid, gut microbiome, inflammasome, LPS

## Abstract

Diet and gut microbiota impact the progression of non-alcoholic steatohepatitis (NASH). An unhealthy diet, rich in fat and sugar, promotes gut microbiota dysbiosis, leading to the translocation of lipopolysaccharides (LPS) and other harmful pathogen-associated molecular patterns to the liver, triggering hepatic inflammation and aggravating NASH. Currently, there are no approved pharmacological treatments for NASH, although novel drugs, such as obeticholic acid (OCA), have demonstrated beneficial effects. Healthy dietary modifications have been proposed to prevent and treat NASH. However, the therapeutic effect of the combined implementation of a healthy dietary intervention with pharmaceutical agents or dietary supplements for NASH treatment and how it interplays with the gut microbiota and associated pathways remains poorly understood. This study aims to investigate the therapeutic efficacy of a healthy dietary modification, either alone or in combination with a dietary supplement [ginger essential oil (GEO)] or drug (OCA), in a Gubra-Amylin diet-induced NASH mouse model. NASH was induced for 12 weeks, after which the interventions were administered for a subsequent 3-week period. Our findings revealed that healthy dietary intervention primarily restores obesity, lipidemia, and NASH. In contrast, GEO and OCA have additional benefits by suppressing pro-inflammatory cytokines by partially modulating the NLRP3/ASC/caspase-1 pathways. Moreover, healthy dietary intervention restores gut microbiota composition and function from dysbiosis, while GEO and OCA partially mediate the gut-derived LPS/toll-like receptor 4/nuclear factor kappa B pathway. Therefore, adherence to healthy dietary patterns plays a primary role in NASH reversion, and additional supplementation of GEO or OCA could offer extra benefits in suppressing hepatic inflammation and preventing disease progression.

**IMPORTANCE:**

The link between gut microbiota and diet is crucial in the development of non-alcoholic steatohepatitis (NASH). This study underscores the essential role of a healthy diet in preventing and treating NASH by reversing obesity, lipidemia, and gut microbiota dysbiosis. Moreover, the supplementation of functional food or drug to the diet can provide additional advantages by inhibiting hepatic inflammation through the modulation of the hepatic inflammasome signaling pathway and partially mediating the gut microbiota and lipopolysaccharide signaling pathway. This study highlights the importance of adopting healthy dietary habits in treating NASH and proposes that supplementing with ginger essential oil or obeticholic acid may offer additional benefits. Nonetheless, further clinical studies are necessary to validate these findings.

## INTRODUCTION

Non-alcoholic fatty liver disease (NAFLD) is a chronic liver condition that affects individuals worldwide (1). NAFLD encompasses a spectrum of liver disorders, including steatosis, non-alcoholic steatohepatitis (NASH), cirrhosis, and hepatocellular carcinoma (2). NASH—characterized by hepatic steatosis with inflammation, ballooned hepatocytes, and hepatic fibrosis—can progress to liver cirrhosis and hepatocellular carcinoma, when untreated. Therefore, early diagnosis of hepatic steatosis can help prevent the development of NASH (3).

Multiple studies have suggested a close association between NAFLD and other metabolic disorders such as obesity, hyperlipidemia, hypertension, and type 2 diabetes (4). One of the major triggers for the development of NAFLD is the consumption of high-fat diet (HFD) (5). An HFD promotes an increase in free fatty acid levels and the accumulation of triglycerides and cholesterol in the liver, with the concomitant production of reactive oxygen species, which can lead to inflammation and cell death (6). HFD can result in the activation of hepatic nucleotide-binding oligomerization domain–leucine-rich repeat (NOD-LRR), and pyrin domain-containing protein 3- (NLRP3) inflammasome, comprising NLRP3, ASC, and pro-caspase-1, which subsequently induce the expression of the inflammatory cytokines interleukin (IL)-1β and -18, yielding liver inflammation and cell death (7, 8).

The theory of multiple hits has frequently been acknowledged regarding the advancement of NAFLD, encompassing various factors such as genetics, unhealthy behaviors, obesity, insulin resistance, and gut microbiota (9, 10). The interaction between gut microbes and diet plays a crucial role in the development of NASH (11). Recent studies have suggested that an unhealthy diet, high in sugar and fat, and low in fiber, leads to imbalanced gut microbiota (12). Furthermore, gut microbiota dysbiosis can generate toxic metabolites such as lipopolysaccharide (LPS), which can be translocated from the gut to the liver (13). Gut-derived bacterial products activate toll-like receptors (TLRs), including TLR4, and trigger downstream inflammatory responses and cytokine generation, enhancing NASH progression. LPS can activate the TLR4 signaling pathway, leading to intracellular signal transduction of the nuclear factor kappa B (NF-κB) and the production of inflammatory cytokines such as tumor necrosis factor (TNF)-α, and IL-1β (14).

Numerous rodent models have been validated for the study of NASH and liver fibrosis. The trans-fat diet mouse model is one of the best models for the induction of NASH (15); however, since the US Food and Drug Administration has banned the use of trans-fat in the food industry, this might not be the most representative model for NASH progression in humans. Recent research substituted the trans-fats with palm oil, the Gubra-Amylin NASH (GAN) diet, an approach resulting in a similar NASH phenotype to that obtained with the trans-fat diet (16). Moreover, liver histological data and transcriptome markers obtained from GAN diet-fed mice are comparable to liver samples from NASH patients (17). The GAN diet also promotes intestinal leakage, endotoxemia, and microbiota dysbiosis, with an increase in harmful bacteria and a decrease in beneficial microbiota (18). Therefore, the GAN diet-fed mouse model represents a suitable approach for investigating the gut microbiota-liver axis.

Various methods have been devised to treat and prevent NASH; however, there are no authorized pharmacological treatments available for the management of NASH. Nevertheless, certain medications, including obeticholic acid (OCA), have displayed favorable outcomes during phase III clinical trials. Nonetheless, some of these drugs encompass several adverse side effects that prevent their clinical application (19, 20). Therefore, uncovering alternative strategies for reversing NASH is crucial. A lifestyle intervention, the combination of diet, exercise, and behavior modification, is an effective and practical approach for managing NAFLD, also improving liver histology in NASH (21). Dietary interventions accompanied by supplementation have been suggested as the most effective approach for improving the inflammatory profile of patients with NAFLD (22). We previously showed that ginger essential oil (GEO) protects the liver from NAFLD by reducing hepatic lipid accumulation, oxidative stress, and inflammation (23, 24). Moreover, GEO also possesses antibacterial properties, which might impact gut microbiota (25, 26). Although this subject has been extensively studied, there is still a gap in the literature regarding the therapeutic effect of the combination of a healthy diet intervention with the use of drugs or dietary supplements in NASH and how this approach interplays with gut microbiota and related pathways.

In this study, we aim to investigate the therapeutic effect of a healthy dietary modification, either alone or in combination with a dietary supplement (GEO) or medication (OCA), on the GAN diet-induced NASH mouse model. We focus on evaluating the impact of these interventions on the gut microbiota function and the LPS/TLR4 and NLRP3 inflammasome pathway.

## RESULTS

### Healthy diet intervention in GAN diet-fed mice ameliorates obesity and lipidemia

**Fig 1:**
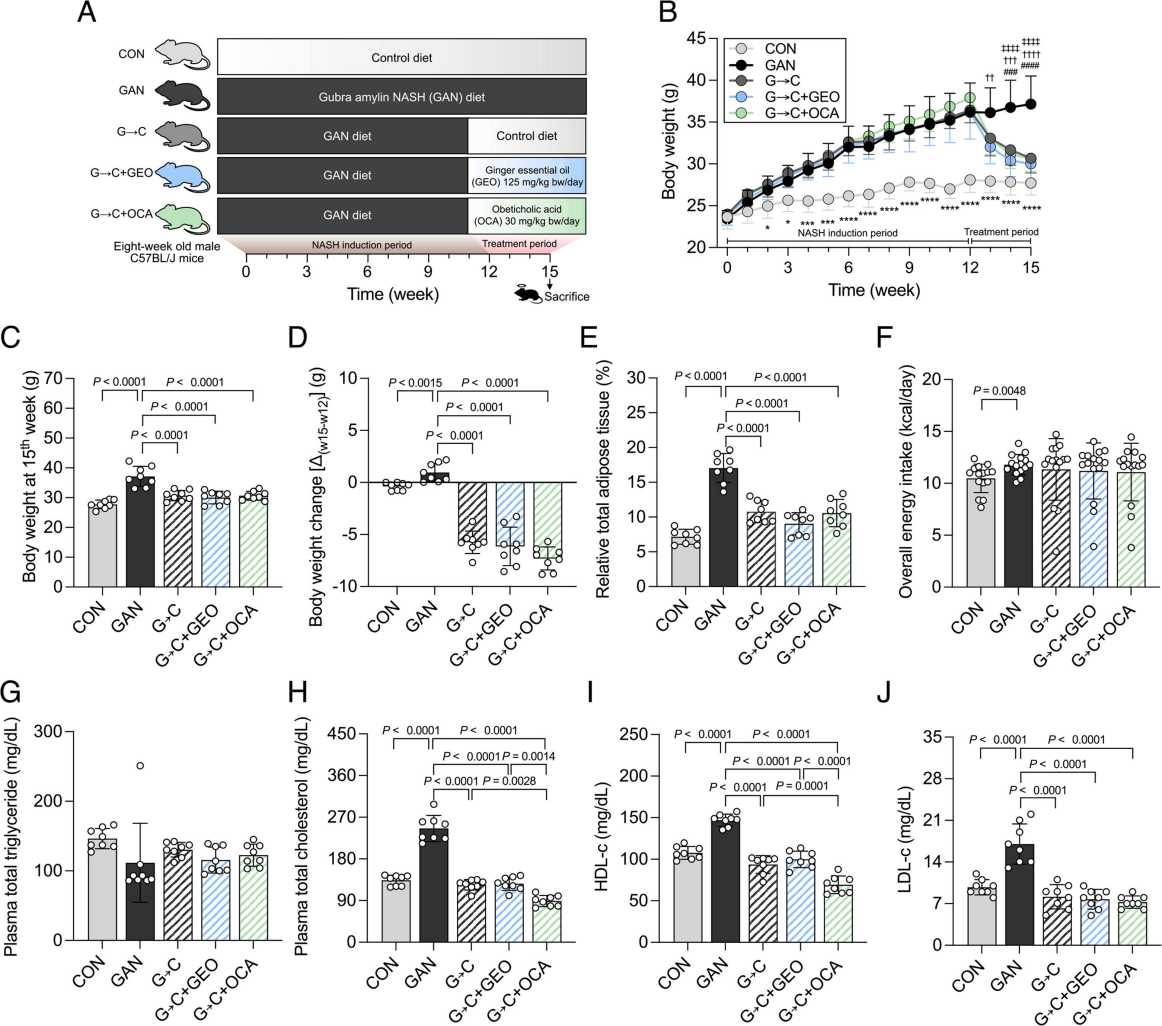
Healthy diet intervention in GAN diet-fed mice alleviates obesity and lipidemia. (**A**) Experimental design, (**B**) body weight change, (**C**) body weight at 15th week, (**D**) body weight change (Δw15 − w12), (**E**) relative total adipose tissue weight, (**F**) overall energy intake, (**G**) plasma total triglyceride, (**H**) total cholesterol, (**I**) high-density lipoprotein cholesterol, and (**J**) low-density lipoprotein cholesterol. C57BL/6 mice were fed with either control or GAN diet for 15 weeks. The mice fed the GAN diet received a healthy control diet, dietary supplement (ginger essential oil; 125 mg/kg bw/day), or drug (obeticholic acid; 30 mg/kg bw/day) intervention by daily oral gavage for 3 weeks. Each value was expressed as the mean ± SD (*n* = 7–8). Statistical analyses were performed by two-tailed student’s *t*-test for comparison between CON and GAN groups (*, *P* < 0.05; **, *P* < 0.01; ***, *P* < 0.001; ****, *P* < 0.0001), and one-way ANOVA with Tukey’s range test for comparisons among GAN diet-fed groups. GAN and G→C groups (#, *P* < 0.05; ##, *P* < 0.01; ###, *P* < 0.001; ####, *P* < 0.0001), GAN and G→C + GEO groups (†, *P* < 0.05; ††, *P* < 0.01; †††, *P* < 0.001; ††††, *P* < 0.0001), GAN and G→C + OCA groups (‡, *P* < 0.05; ‡‡, *P* < 0.01; ‡‡‡, *P* < 0.001; ‡‡‡‡, *P* < 0.0001). G→C, change GAN to CON diet; G→C + GEO, change diet with GEO supplement; and G→C + OCA, change diet with OCA.

Our experimental design, depicted in Fig. 1A, aimed to investigate the therapeutic effects of combining a healthy diet intervention with a drug (OCA) or dietary supplement (GEO) on NASH. Male C57BL/6 J mice were allocated to either the control group or the GAN diet-fed group, where the latter diet served as a NASH-inducer over a period of 12 weeks. After this period, the control group was maintained in the control diet (CON), while the GAN-fed group was randomly divided into four subgroups: (i) continued to receive the GAN diet (GAN); (ii) switched to the control diet (G→C); (iii) switched to the control diet and given GEO supplementation (125 mg/kg bw/day) (G→C + GEO); and (iv) switched to the control diet and given OCA supplementation (30 mg/kg bw/day) (G→C + OCA), administered through daily oral administration for 3 weeks. The GAN diet comprises 40 kcal% non-trans-fat (mainly palm oil), 20% fructose, and 2% cholesterol. The animal model used in the experiment was previously shown to induce NASH after being fed the GAN diet for 12 weeks (18), and the dosages of GEO and OCA were based on previous studies (24, 27, 28). Mice in the GAN group developed significant obesity with a gradual increase in body weight from the second week of GAN diet ingestion compared to the CON group (*P* < 0.05). Other parameters such as the final body weight (15th week, *P* < 0.0001), relative total adipose tissue (*P* < 0.0001), and overall energy intake (*P* < 0.0048) were significantly higher in the GAN group (Fig. 1B through F). After switching the GAN diet to the control diet for 3 weeks, the G→C, G→C + GEO, and G→C + OCA groups showed a rapid reduction in body weight compared to the GAN group (*P* < 0.0001). Specifically, body weight reduction was 5.7, 6.1, and 7.3 g (*P* < 0.0001) in the G→C, G→C + GEO, and G→C + OCA groups, respectively, which accounted for a 18.7%, 20.5%, and 23.9% reduction of the body weight upon the 3-week intervention (Fig. 1C and D). The relative total adipose tissue was also significantly reduced after these interventions (*P* < 0.0001), as observed in all types of adipose tissue, including epididymal white adipose tissue (WAT), inguinal WAT, perirenal WAT, mesenteric WAT, inguinal subcutaneous white adipose tissue, and interscapular brown adipose tissue (iBAT) (Fig. 1E and Fig. S2). We observed no significant differences among the G→C, G→C + GEO, and G→C + OCA groups regarding body weight, relative adipose tissue, or overall energy intake (Fig. 1F), indicating that the healthy diet intervention was the primary factor in ameliorating obesogenic biomarkers. Plasma triglyceride levels were similar among groups (Fig. 1G); however, the GAN group showed increased plasma total cholesterol (TC), high-density lipoprotein cholesterol (HDL-c), and low-density lipoprotein cholesterol (LDL-c) (*P* < 0.0001) compared to the CON group, whereas the G→C, G→C + GEO, and G→C + OCA interventions significantly decreased these parameters (Fig. 1H through J), suggesting that dietary changes strongly reduced blood lipid levels. The mice in G→C + OCA group exhibited more pronounced reductions in total cholesterol and HDL-c compared to the G→C group (*P* < 0.0028 and *P* < 0.0001, respectively). These results confirm that a healthy diet intervention primarily ameliorates obesity and lipidemia, while the addition of OCA plays a secondary role in the reduction of plasma lipid levels.

### Healthy diet intervention ameliorates NASH

**Fig 2:**
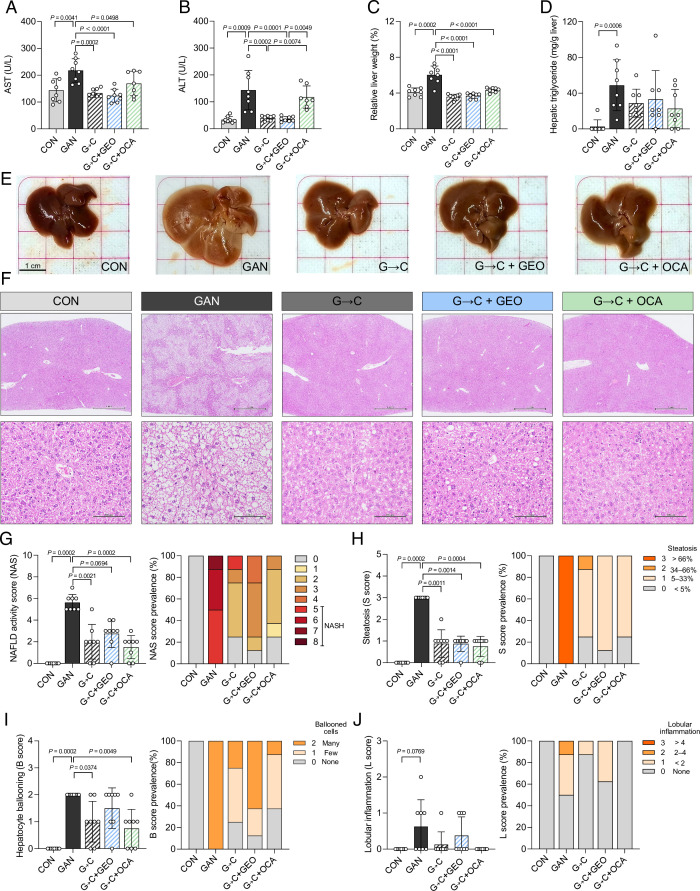
Healthy diet intervention in GAN diet-fed mice alleviates NASH demonstrated by the improvement of plasma hepatic damage biomarkers and reversal of the NAFLD activity, steatosis, and hepatocyte ballooning scores. (**A**) Plasma AST, (**B**) ALT, (**C**) relative liver weight, (**D**) hepatic triglyceride, (**E**) representative of liver images, (**F**) liver histopathological changes, (**G**) NAFLD activity score, (**H**) steatosis score, (**I**) hepatocyte ballooning score, and (**J**) lobular inflammation score and their prevalence. The mice fed the GAN diet received a healthy control diet, dietary supplement (ginger essential oil; 125 mg/kg bw/day), or drug (obeticholic acid; 30 mg/kg bw/day) intervention by daily oral gavage for 3 weeks. Each value was expressed as the mean ± SD (*n* = 7–8). Statistical analyses were performed by a two-tailed Student’s *t*-test for comparison between CON and GAN groups (Wilcoxon signed-rank test for liver histology score), and one-way ANOVA with Tukey’s range test for comparisons among GAN diet-fed groups (Kruskal-Wallis tests with Dunn’s multiple comparisons for liver histology score). G→C, change GAN to CON diet; G→C + GEO, change diet with GEO supplement; and G→C + OCA, change diet with OCA.

The use of blood aspartate aminotransferase (AST) and alanine aminotransferase (ALT) as biomarkers helps monitor liver damage, and an increase in liver weight can indicate fat accumulation in the liver. The mice in the GAN group exhibited significantly elevated levels of AST, ALT, relative liver weight, and hepatic triglyceride compared to the CON group (*P* = 0.0041, *P* = 0.0009, *P* = 0.0002, and *P* = 0.0006) (Fig. 2A through D). The 3-week interventions with G→C, G→C + GEO, and G→C + OCA promoted a reduction in AST and relative liver weight, while ALT levels were reduced only in the first two groups. Furthermore, the interventions promoted a trend for decreased hepatic triglyceride (Fig. 2D). Representative macroscopic images of the liver (Fig. 2E) showed that GAN diet resulted in an increase in liver size and the whitening of the tissue, whereas the 3-week interventions reverted these observations. Liver histological analysis revealed higher lipid droplet accumulation in the GAN group compared to those in CON group or those receiving healthy diet interventions with or without GEO and OCA supplementation (Fig. 2F). The NAFLD activity score (NAS) and scores for steatosis, hepatocyte ballooning, and lobular inflammation were also assessed. A NAS ≥5 indicates NASH (Fig. 2G through J), and GAN diet feeding resulted in a NASH prevalence of 100% (*P* = 0.0002, compared to CON group). The groups receiving interventions with G→C, G→C + GEO, and G→C + OCA exhibited a reduction in NAS scores (*P* = 0.0021, *P* = 0.0694, and *P* = 0.0002, respectively) and a decreased NASH prevalence of 12.5%, 0% and 0%, respectively, indicating that supplementation with GEO and OCA helped alleviate NASH prevalence better than dietary intervention alone. The interventions improved steatosis scores, and G→C and G→C + OCA groups exhibited decreased hepatocyte ballooning scores (Fig. 2H and I). Lobular inflammation was also reduced in all interventions, although not statistically significant (Fig. 2J). Overall, these findings suggest that healthy diet intervention in GAN diet-fed mice ameliorates NASH, shown by the decrease of plasmatic hepatic damage biomarkers and the reversion of the NAFLD activity, steatosis, and hepatocyte ballooning scores.

### Supplementation with GEO and OCA demonstrated additional beneficial effects on inflammation

**Fig 3:**
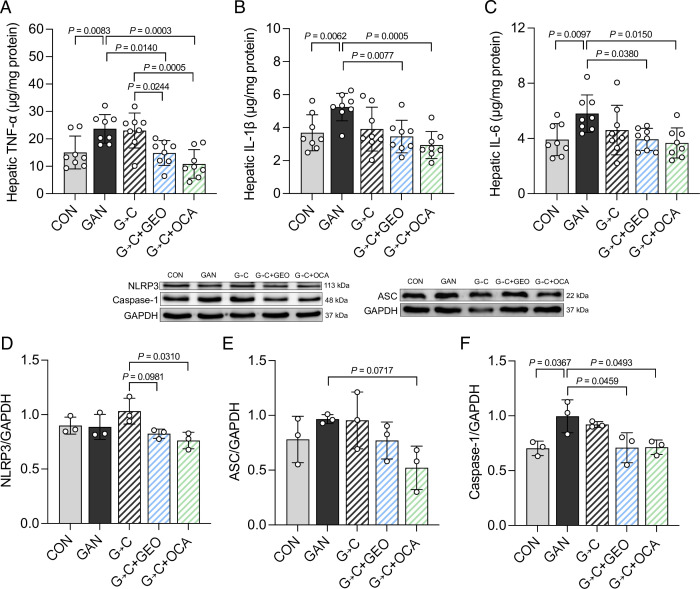
Supplementation with GEO and OCA suppresses inflammatory cytokines and modulates the NLRP3/ASC/caspase-1 pathways more robustly than healthy diet alone. (A) Liver TNF-α, (B) IL-1β, (C) IL-6, (D) NLRP3, (E) ASC, and (F) caspase-1. Each value was expressed as the mean ± SD [pro-inflammatory cytokine data (*n* = 7–8); protein expression data (*n* = 3). Statistical analyses were performed by a two-tailed Student's *t*-test for comparison between CON and GAN groups, and one-way ANOVA with Tukey's range test for comparisons among GAN diet-fed groups. G→C, change GAN to CON diet; G→C + GEO, change diet with GEO supplement; and G→C +OCA, change diet with OCA.

Since inflammation is critical for the progression of NASH, we evaluated the levels of hepatic pro-inflammatory cytokines. The GAN group exhibited increased pro-inflammatory cytokines such as TNF-α (*P* = 0.0083), IL-1β (*P* = 0.0062), and IL-6 (*P* = 0.0097). The intervention of G→C alone did not impact the levels of hepatic pro-inflammatory cytokines (Fig. 3A through C); however, the G→C + GEO and G→C + OCA interventions significantly decreased TNF-α (*P* = 0.0140 and *P* = 0.0003), IL-1β (*P* = 0.0077 and *P* = 0.0005), and IL-6 (*P* = 0.0380 and *P* = 0.0150) compared to the GAN group and the G→C group. We also evaluated the hepatic levels of NLRP3 inflammasome-related proteins, including NLRP3, ASC, and caspase-1. GAN feeding had no impact on NLRP3 protein expression compared to the CON group; however, the G→C + GEO and G→C + OCA interventions substantially decreased NLRP3 protein expression compared to the G→C group (*P* = 0.0981 and *P* = 0.0310) (Fig 3D). The protein levels of ASC were not significantly different, except in the G→C + OCA group, which exhibited a substantial reduction (*P* = 0.0717) (Fig. 3E). The GAN diet significantly increased caspase-1 protein levels compared to the CON group (*P* = 0.0367), which was significantly reverted with the G→C + GEO and G→C + OCA interventions (*P* = 0.0459; *P* = 0.0493) (Fig. 3F). The supplementation of GEO and OCA in the G→C intervention promotes further beneficial effects through the decrease in pro-inflammatory cytokines which might be mediated through the NLRP3/ASC/caspase-1 pathways.

### Healthy diet intervention remodels gut microbiota composition; GEO and OCA play a secondary impact compared to diet intervention

**Fig 4:**
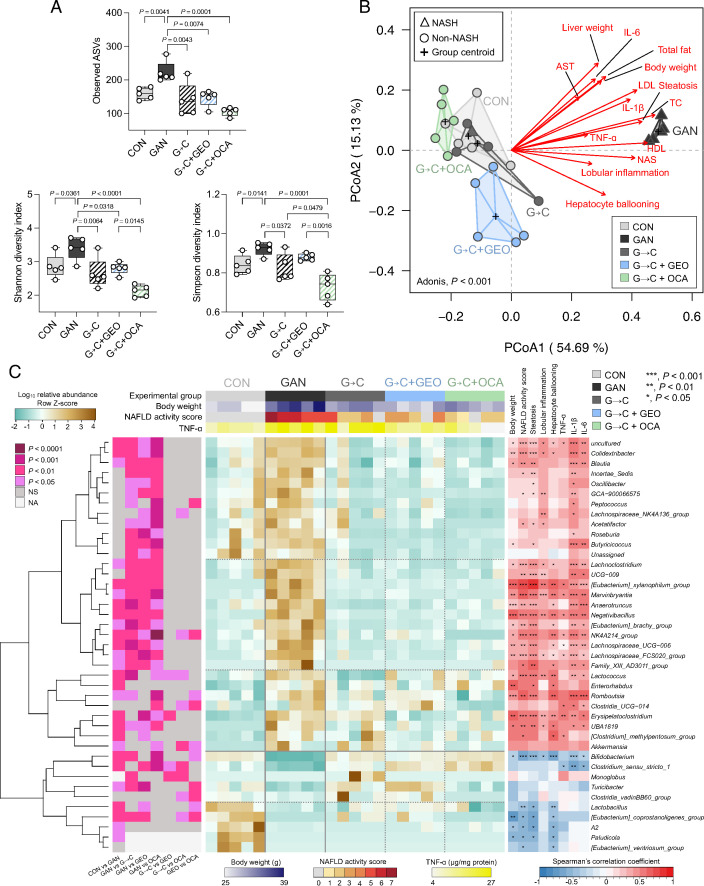
Healthy diet intervention reversed GAN-induced gut microbiota alterations and remodeled gut microbiota composition. (A) α-Diversity indices, observed ASVs, Shannon index, and Simpson diversity index; (B) principal coordinate analysis (PCoA) plot based on Bray-Curtis dissimilarity with biomarker vector; (C) heatmap of the relative abundances of fecal microbiota with a significant difference measured using the Kruskal-Wallis test (*P* < 0.05) level and Spearman’s correlation analysis between gut microbiota at genera and obesity-, NAFLD-, and hepatic inflammation-associated biomarker. Each value was expressed as the mean ± SD (*n* = 5). Statistical analyses were performed by a two-tailed Student's *t*-test for comparison between CON and GAN groups, and one-way ANOVA with Tukey's range test for comparisons among GAN diet-fed group. Analysis of variance using distance matrices (Adonis) was calculated to determine the heterogeneity of the feces microbiota among the groups in PCoA. Vectors in the PCoA plot indicated a significant effect of biomarkers (*P* < 0.05), and its length shows the strength of the correlation. Statistical analyses in heatmap were performed using an unpaired Wilcoxon signed-rank test; and Kruskal-Wallis test with Dunn’s multiple comparisons test for comparing among GAN diet-fed group. G→C, change GAN to CON diet; G→C + GEO, change diet with GEO supplement; and G→C + OCA, change diet with OCA.

Gut microbiota plays a critical role in the pathogenesis of NASH; therefore, to investigate the impact of healthy diet intervention and of GEO and OCA, we sequenced the V3-V4 16S rRNA gene from fecal samples collected from the colon. The bioinformatics pipeline generated an amplicon sequence variant (ASV) table aligned against the SILVA database (version 138). A total of 598 ASVs were identified and assigned to 166 genera. The α-diversity was determined using the observed ASVs and the Shannon and Simpson indices from the ASV table. The ASV, Shannon, and Simpson diversity indices were significantly increased in the GAN group compared to the CON group (*P* = 0.0041, *P* = 0.0361, and *P* = 0.0141, respectively) (Fig. 4A), suggesting that GAN diet increased the richness and the evenness of the gut microbiota community. The G→C, G→C + GEO, and G→C + OCA groups exhibited a reduction of the three α-diversity indices, to levels similar to the CON group (Fig. 4A). Additionally, the G→C + OCA treatment led to a reduction in both Shannon and Simpson diversity indices compared to G→C + GEO. However, only the Simpson diversity index was significantly lower when compared to the G→C group, indicating that OCA supplementation selectively reduced the evenness of the gut microbiota composition. We further assessed the β-diversity based on the Bray-Curtis distance and observed significantly different gut microbiota among groups (Adonis; *P* < 0.001) (Fig. 4B). Diet was identified as the primary factor in the shift of gut microbiota in PCoA1 (54.69%), while supplementation of GEO and OCA played a secondary role in PCoA2 (15.13%). The GAN group was distinctly separated from the CON, G→C, G→C + GEO, and G→C + OCA groups in PCoA plot. Among the CON and G→C clusters, the G→C + GEO and G→C + OCA were found separated from the G→C group, suggesting that GEO and OCA in G→C shifted gut microbiota composition. In contrast, the G→C group was unseparated from the CON group, indicating that G→C intervention could reverse the gut microbiota composition, closer to the CON group. The association between NASH-related features and gut microbiota composition was assessed and shown as a vector. The results showed a significant association (*P* < 0.05) between NASH- and obesity-related parameters and the gut microbiota of GAN-fed mice, including body weight, total fat, TC, LDL, AST, liver weight, NAS, steatosis, lobular inflammation, hepatocyte ballooning, TNF-ɑ, IL-1β, and IL-6.

Heatmap represented differed 41 genera using the Kruskal-Wallis test (*P* < 0.05) (Fig. 4C). The heatmap displayed the two primary hierarchical clustering of the gut microbiota, suggesting that the diet type was the primary factor differentiating the fecal microbiota at the genus level. The GAN group cluster consisted of 31 genera-enriched microbiota, while the control group cluster consisted of 10 genera, and G→C, G→C + GEO, and G→C + OCA were found in a subcluster within the control diet-enriched microbiome cluster, indicating that they played a secondary role in altering the gut microbiota. The top panel of the heatmap showed the experimental groups, body weight, NAS, and TNF-ɑ; these biomarkers were increased in the GAN group but reduced in CON, G→C, G→C + GEO, and G→C + OCA group. Pairwise statistical analyses were performed using an unpaired Wilcoxon signed-rank test, and the results were displayed in the left panel of the heatmap with varying *P*-values.

The GAN group exhibited a significant increase in the relative abundance of bacteria associated with disease, obesity, or NAFLD compared to the CON group, including *Blautia*, *Lachnoclostridium*, [*Eubacterium]_xylanophilum_group*, *Anaerotruncus*, *Negativibacillus*, *Lachnospiraceae_UCG−006*, *Lachnospiraceae_FCS020_group*, *Romboutsia*, *Erysipelatoclostridium*, as well as other genera such as *Colidextribacter*, *Marvinbryantia*, [*Eubacterium*]_*brachy*_*group*, *NK4A214*_group, *Family*_*XIII*_*AD3011*_*group*, *Lactococcus*, *Enterorhabdus*, *UBA1819*, [*Clostridium*]_*methylpentosum*_*group*, and *Akkermansia*. Conversely, the GAN diet group exhibited a decrease in beneficial bacteria such as *Bifidobacterium*, *Turicibacter*, *Lactobacillus*, and other genera, including *Clostridium*_*sensu*_*stricto*_*1*, [*Eubacterium*]_*coprostanoligenes*_*group*, *A2*, and *Paludicola*. Using Spearman’s correlation analysis, we observed a significant positive correlation between the group of genera enriched in the GAN diet and obesity and biomarkers associated with non-alcoholic steatohepatitis, while the cluster of genera depleted by the GAN diet exhibited a significant negative correlation with these health-promoting genera. The G→C intervention reversed the GAN-induced changes to the gut microbiota, resulting in a composition similar to that of the control group, except for certain genera, such as *A2*, *Paludicola*, and [*Eubacterium*]_*ventriosum*_*group*. The introduction of additional supplementation of GEO and OCA had a limited impact on the gut microbiota at the genus level, resulting in the modification of only four and nine genera for GEO and OCA, respectively (Fig. 4C, left panel). However, the impact of GEO and OCA on the gut microbiota is distinct, with 10 genera exhibiting significant differences between the G→C + GEO and G→C + OCA groups. These findings suggest that the healthy diet intervention has a primary effect on the gut microbiota composition, and the supplementation of GEO and OCA has a secondary impact.

### The 3-week interventions directly impacted gut microbiota function via the LPS/TLR4/NF-κB signaling pathway

**Fig 5:**
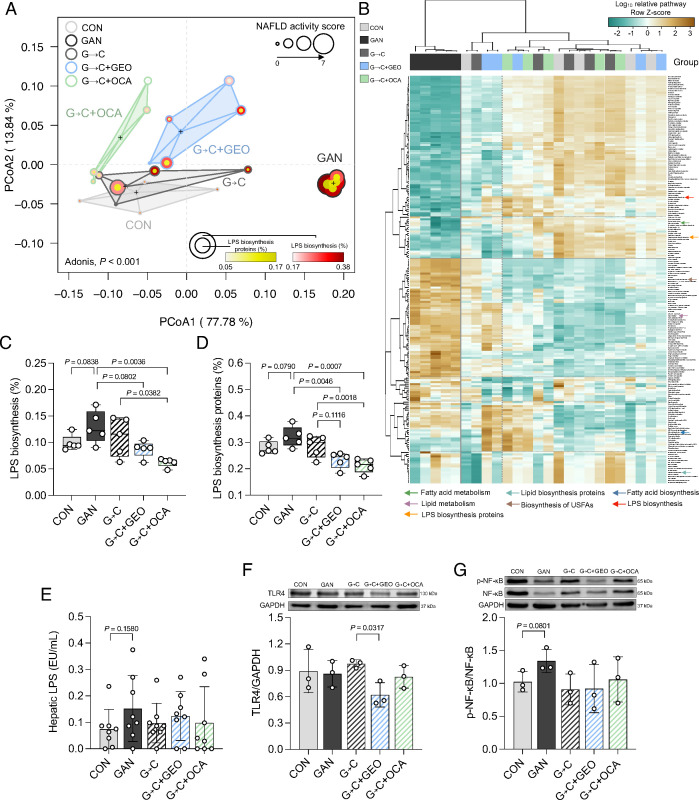
Healthy diet intervention, GEO, and OCA impact gut microbiota function and modulate the gut-derived LPS/TLR4/NF-κB signaling pathway. (A) Principal coordinate analysis (PCoA) plot based on Bray-Curtis dissimilarity of the gut microbiome PICRUSt2 functional prediction, (B) heatmap of the gut microbiota functional capabilities prediction based on the KEGG database with a significant difference measured using the Kruskal-Wallis test with FDR-adjusted *P*-value (*P* < 0.05), (C) LPS biosynthesis, (D) LPS biosynthesis proteins function, (E) hepatic LPS, (F) TLR4, and (G) p-NF-κB/NF-κB protein expression. Each value was expressed as the mean ± SD [gut microbiota function data (*n* = 5); protein expression (*n* = 3)]. Statistical analyses were performed by a two-tailed Student’s *t*-test for comparison between CON and GAN groups, and one-way ANOVA with Tukey’s range test for comparisons among GAN diet-fed group. Analysis of variance using distance matrices (Adonis) was calculated to determine the heterogeneity of the feces microbiota function among the groups in PCoA. Statistical analyses in heatmap were performed using Kruskal-Wallis test. G→C, change GAN to CON diet; G→C + GEO, change diet with GEO supplement; and G→C + OCA, change diet with OCA.

The functional prediction of gut microbiota was conducted using the phylogenetic investigation of communities by reconstruction of unobserved states (PICRUSt2), generating 320 pathways. A PCoA plot of gut microbiota function showed a similar separation to that of gut microbiota composition, with PCoA1 and PCoA2 explaining 77.78% and 13.84% of the variation, respectively (Fig. 5A). The dietary intervention, GEO, and OCA significantly affected the gut microbiota function compared to the GAN group, as determined by Adonis (*P*-value of 0.001; Fig. 5A). The PCoA plot also revealed information on the NAS score, LPS biosynthesis, and protein functions, where larger dot plots and deeper colors indicate an increase in these parameters, more prominent in the GAN group. Using the Kruskal-Wallis test with FDR-adjusted *P*-values (*P* < 0.05), we observed 203 functions (relative abundance >0.001%) significantly distinct among the groups, namely fatty acid metabolism, lipid biosynthesis proteins, fatty acid biosynthesis, lipid metabolism, biosynthesis of unsaturated fatty acids, LPS biosynthesis, LPS biosynthesis proteins, etc. (Fig. 5B). The GAN group showed a trend for increased LPS biosynthesis and LPS biosynthesis proteins (Fig. 5C and D), what was reverted in the G→C + GEO and G→C + OCA groups.

The presence of dysfunctional intestinal barriers allows the entry of gut microbial LPS into the circulatory system and the liver, which promotes inflammation. With our TLR4/NF-κB/secreted embryonic alkaline phosphatase (SEAP) reporter HEK293 cell assay, we observed a reduction trend of LPS levels in the groups treated with G→C, G→C + GEO, and G→C + OCA (Fig. 5E). These data suggest that switching the diet with or without GEO and OCA supplementation promotes beneficial improvements in gut microbiota function by downregulating LPS-related pathways.

Mechanistically, gut-derived LPS activates the TLR4/NF-κB pathway, leading to hepatic inflammation. We examined the hepatic protein expression of TLR4 and p-NF-κB/NF-κB and found that only G→C + GEO was able to reduce TLR4 protein expression, while G→C, G→C + GEO, and G→C + OCA tended to reduce p-NF-κB/NF-κB protein expression (Fig. 5F and G). Our data demonstrate that intervention with a healthy diet, GEO, and OCA impacts gut microbiota function and partially modulates the gut microbiota and its metabolite-liver axis through the LPS/TLR4/NF-κB signaling pathway.

## DISCUSSION

NASH is a critical and increasingly common liver disease without any approved pharmacological treatments; therefore, developing alternative strategies for managing and reversing NASH is key. Lifestyle interventions that combine diet, exercise, and behavior modification are practical and efficient in the management of NASH. Furthermore, several dietary approaches have been suggested to target the incidence and severity of NAFLD, such as the Mediterranean, high-protein, hypocaloric, high-carbohydrate/low-fat, low-carbohydrate/high-fat, and intermittent calorie restriction diets (29). Nonetheless, the therapeutic effect of combining a healthy diet intervention with drugs or dietary supplements on NASH and how this approach impacts the gut microbiota and its related pathways remains to be understood. Our study demonstrates the potential therapeutic effect of a healthy diet modification, either alone or combined with a dietary supplement (GEO) or medication (OCA), on a GAN diet-induced NASH mouse model. The GAN diet is an emerging innovative approach to generating rodent NASH models that can recapitulate the human NASH phenotype, resulting in morphological characteristics similar to those observed in human NASH (17). Additionally, the GAN diet increases intestinal permeability, endotoxemia, and dysbiosis of the intestinal microbiota, by promoting the growth of pathogenic bacteria and suppressing beneficial microbiota (18). Here, we demonstrate that the GAN diet-fed mice develop significant obesity and lipidemia. A study with a large cohort found that patients with NASH display dyslipidemia, namely high serum levels of total triglycerides (TG), TC, and LDL-c and lower levels of HDL-c (30). A previous study reported that an intervention using chow diet intervention in the GAN diet-induced obese mice for 8 and 12 weeks substantially improves metabolic outcomes and liver histology (31). In our study, a 3-week transition to a healthy control diet, with or without GEO or OCA supplementation, promoted to a rapid reduction in body weight and adipose tissue, and modulation of dyslipidemia. We observed that the healthy diet intervention was the primary factor in ameliorating obesogenic biomarkers, while the addition of OCA played a synergistic role, further reducing plasma lipid levels. In GAN-fed mice, we observed an increase in HDL-c levels, which may be attributed to the consumption of palm oil, a saturated fat that has been shown to elevate HDL-c (32). However, in the OCA group, there was a significant reduction in HDL-c, which has already been described as a known side effect (33).

In this study, the consumption of the GAN diet increased liver damage biomarkers, such as AST, ALT, liver weight, and hepatic triglyceride, compared to the control group. The GAN diet is constituted by high levels of palm oil, fat, fructose, and cholesterol. Prior research has linked dietary consumption of saturated fat and fructose to intrahepatic lipid accumulation, lipogenesis, insulin resistance, oxidative stress, and inflammation (30, 34). Palm oil is rich in saturated fatty palmitic acid (C16:0) (35), which can disrupt metabolic and glucose homeostasis and induce inflammation in the liver and white adipose tissue, both normocaloric and normolipidic diets. These adverse effects are more pronounced in mice treated with interesterified palm oil (36). Here, the groups that underwent healthy diet intervention, either alone or in conjunction with GEO or OCA, exhibited a decrease in these biomarkers and an improvement in liver histology scores for NAFLD activity, steatosis, and hepatocyte ballooning. This study emphasizes the crucial role of a healthy diet in preventing and treating liver disease, particularly NASH. Additionally, the supplementation with GEO and OCA improved the efficiency of dietary intervention alone. Nonetheless, OCA was unable to decrease ALT levels, which could be attributed as a side effect.

Our study uncovered that dietary intervention alone improved the NAS score by reducing inflammation, without changing the levels of pro-inflammatory cytokines; however, the supplementation with GEO and OCA significantly reduced of pro-inflammatory cytokines (TNF-ɑ, IL-1β, and IL-6). Previous studies have demonstrated that ginger essential oil offers protective effects against non-alcoholic fatty liver disease by mitigating hepatic lipid accumulation, oxidative stress, and inflammation (23, 24). Moreover, GEO is also recognized for its antibacterial properties, which might influence the composition and function of the gut microbiota (25, 26). GEO has been shown to have anti-inflammatory effects, significantly reducing the severity of acetic acid-induced colitis in rats. Furthermore, the main component of GEO, citral, was shown to reduce LPS-induced systemic inflammation in obese mice (37, 38). NLRP3 inflammasome plays a critical role in NASH and liver inflammation (39). We observed a trend for decreased levels of NLRP3, ASC, and caspase-1 in the groups receiving GEO and OCA, suggesting that this supplementation might have a stronger effect than dietary intervention alone, not only in the inhibition of inflammatory cytokines but also in inflammasome. GEO has been reported to prevent NASH progression by blockading the NLRP3 inflammasome pathway and mediating hepatic pro-inflammatory cytokines (24). OCA has been found to inhibit NLRP3 inflammasome activation in macrophages, suggesting a new mechanism for treating NASH (40).

Gut microbiota plays a substantial role in the pathogenesis of NASH. Alterations in the composition and function of the gut microbiota have been observed in patients with NASH, including an increase in potentially pathogenic bacteria and a decrease in beneficial bacteria (41). Gut microbiota can affect liver function by producing harmful metabolites that can cause inflammation, leading to the development of NASH (14). Here, we show that a healthy diet intervention remodeled and reversed the gut microbiota composition in both ɑ- and β-diversity indices, while the OCA and GEO had a minor impact compared to the diet intervention. The GAN diet increased the richness and the evenness of the gut microbiota community. The intervention with OCA decreased Shannon and Simpson diversity indices, suggesting an impact on the gut microbiota composition evenness. We also found that the GAN-fed group had a significant increase in the relative abundance of bacteria associated with disease, obesity, or NAFLD, including *Blautia*, *Lachnoclostridium*, [*Eubacterium*]_*xylanophilum*_*group*, *Anaerotruncus*, *Negativibacillus*, *Lachnospiraceae*_*UCG−006*, *Lachnospiraceae*_*FCS020*_*group*, *Romboutsia*, and *Erysipelatoclostridium*. Conversely, this group also exhibited a decrease in beneficial bacteria such as *Bifidobacterium*, *Turicibacter*, and *Lactobacillus*. Moreover, Spearman’s correlation analysis revealed the consensus with respect to the clusters of genera that were either enriched or depleted in the GAN group.

The genus *Blautia* has been found to be abundant in NAFLD patients with NASH (42), while *Lachnoclostridium* has been associated with obesity (43). In mice, the [*Eubacterium*]_*xylanophilum*_*group* and *Erysipelatoclostridium* are enriched by a Western diet (44), while *Anaerotruncus* increases with a high-cholesterol diet (45). *Negativibacillus sp000435195* shows a strong positive association with a high fatty liver index (46). *Lachnospiraceae*_*UCG−006* is enriched in mice fed a high-fat diet, and *Lachnospiraceae*_*FCS020*_*group* is associated with the absence of lymph node metastasis in colorectal cancer (47, 48). *Romboutsia* is significantly associated with the progression of NASH and fibrosis when combined with type 2 diabetes (49). *Bifidobacterium adolescentis* and *Lactobacillus rhamnosus* were found to alleviate HFD-high-cholesterol-induced NAFLD through modulation of gut microbiota-dependent pathways (50), while *Bifidobacterium lactis* V9 attenuates liver steatosis and inflammation in rodents with NAFLD (51). *Turicibacter* was found to be more abundant in lean than in obese rodents and may have anti-inflammatory properties (52). *Lactobacillus* is a probiotic that improves steatohepatitis through the gut microbiota-liver axis by modulating microbiota composition and inflammation in NAFLD (53). *Lactobacillus casei* Shirota was found to prevent fructose-induced liver steatosis through the hepatic TLR4 signaling cascade (54). Administration of *Lactobacillus rhamnosus* GG in fructose-induced NAFLD mice reduces portal LPS, restores gut barrier function, and attenuates liver inflammation and steatosis (55). The G→C intervention reverted the GAN-induced alterations to the gut microbiota, resulting in a composition similar to that of the control group. Supplementation of GEO and OCA showed a limited effect on the gut microbiota at the genus level, modifying only a few genera.

Beyond gut microbiota composition, the dietary intervention, GEO, and OCA also impacted the gut microbiota function, mostly related to fat metabolism. Interestingly, LPS biosynthesis and its proteins increased in the GAN group and decreased in the G→C + GEO and G→C + OCA groups. Dysfunctional intestinal barriers allow gut microbial LPS to enter the liver and circulatory system, promoting inflammation (56). The healthy diet intervention, with or without GEO and OCA supplementation, tended to reduce LPS in the liver which corresponded with gut microbiota function data. Studies have reported an increase in LPS within hepatocytes in patients with NAFLD, which may initiate liver inflammation through TLR4-associated pathways (57). Mechanistically, LPS derived from the gut activates the TLR4/NF-κB pathway, leading to hepatic inflammation (58). Beyond the activation of the TLR4/NF-κB pathway, LPS can also activate the NLRP3 inflammasome (59). In this study, we found that only the G→C + GEO intervention was able to reduce TLR4 protein levels, while there was a tendency for reduced p-NF-κB/NF-κB levels in the dietary intervention with or without GEO or OCA groups. Therefore, we hypothesize that the intervention with a healthy diet, GEO, and OCA impacts gut microbiota composition and function partially through the LPS/TLR4/NF-κB signaling pathway.

The experimental design of this study, which involved combining the treatment with a healthy diet and GEO or OCA, was motivated by our intention to mirror the typical clinical scenario where, upon diagnosis with NASH, patients are often advised by healthcare professionals to adopt healthier dietary habits and may be prescribed medications or dietary supplements. However, this study did not evaluate the effect of supplementing with GEO or OCA alone (without dietary change), which would have allowed us to discern whether the diet has a confounding impact on these supplements. In future experiments, separate the individual effects of GEO and OCA, which need to be evaluated. Furthermore, in our study, we conducted a comparative analysis of gut microbiota composition after transitioning to the altered diet with or without GEO and OCA. However, we recognize that this design may not fully capture the more immediate and progressive changes in the microbiota in response to these dietary modifications. Understanding the temporal dynamics of gut microbiota changes requires a more longitudinal perspective. Conducting weekly gut microbiota analyses could provide a better understanding of how the gut microbiota and the host react to these dietary shifts in composition and function.

### Conclusion

**Fig 6:**
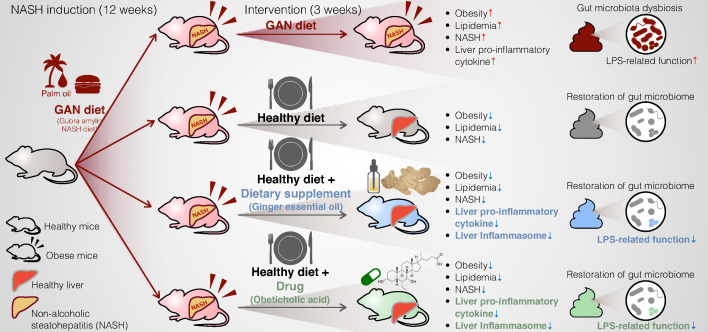
The progression of NASH can be reversed through gut microbiota modulation by a healthy diet intervention, with or without the addition of GEO and OCA. In NASH mice, a healthy diet intervention serves as the primary intervention for restoring obesity, lipidemia, steatohepatitis, and gut microbiota composition and function. The supplementation of functional food (GEO) and drug (OCA) demonstrates additional advantages by inhibiting pro-inflammatory cytokines and improving hepatic inflammation through the NLRP3/ASC/caspase-1 pathway, as well as reducing gut microbiota LPS-related function via LPS/TLR4/NF-κB.

This study shows that a 3-week period of healthy diet intervention, with or without GEO and OCA supplementation, has a restorative effect on NASH, obesity, and lipidemia. The dietary intervention has a primary role in the amelioration of NASH, whereas the combined supplementation of GEO and OCA with a healthy diet intervention demonstrated additional benefits through the inhibition of hepatic pro-inflammatory cytokines mediated through the NLRP3/ASC/caspase-1 pathways. The healthy diet intervention was found to remodel and reverse the gut microbiota composition and function, with GEO and OCA having a minor impact. Moreover, these interventions partially impacted the LPS/TLR4/NF-κB signaling pathway, a determinant for NASH development (Fig. 6). In conclusion, the combination of a dietary supplement (GEO) or drug (OCA) showed a favorable effect on NASH treatment. However, it is required to validate this effect in a clinical study.

## MATERIALS AND METHODS

### Animal model

Six-week-old C57BL/6J male mice were obtained from the Taiwan National Laboratory Animal Center. They were housed in an animal room with a controlled temperature of 21 ± 2°C, a relative humidity of 60 ± 10%, and 12-h light/dark cycles, without any intervention for an adaptation period of 2 weeks. They were then randomly divided into two groups: one group was fed a control diet (10 kcal% fat and carbohydrate mainly as corn starch, Research Diets, Inc., NJ, USA; D12450K), and the other a GAN diet (Gubra-Amylin NASH diet; 40% total fat kcal, 20% fructose, 2% cholesterol; Research Diets, Inc., NJ, USA; D09100310) (17) for 12 weeks to induce NASH (18). At the end of 12 weeks, the CON group was fed the control diet (*n* = 8), whereas the GAN group was randomly divided into four subgroups (*n* = 7–8/group): (i) GAN group, which continued to receive the GAN diet; (ii) G→C group, which was switched to the control diet; (iii) G→C + GEO group, which was switched to the control diet and oral administration of GEO supplementation by gavaging (125 mg/kg bw/day) (24); (iv) G→C + OCA group, which was switched to the control diet along with oral OCA supplementation (30 mg/kg bw/day) (27, 28). This intervention was conducted for an additional 3 weeks. Body weight and food intake were measured weekly throughout the study. After the intervention (12 + 3 weeks), mice were euthanized using carbon dioxide asphyxiation, and blood was extracted through cardiac puncture with a heparin-coated syringe. The liver and adipose tissue were immediately collected, as well as the fecal content from the colon.

### GEO extraction and ingredient analysis

The ginger essential oil extraction was performed as previously described (23 – 25). Briefly, ginger (*Zingiber officinale* Roscoe) was purchased from Nantou County, Taiwan and mixed with water in a 1:3 ratio) using a blender. The mixture was distilled for 5–6 h through steam distillation to obtain GEO. GEO was frozen to remove water two to three times and stored at −20℃. For the analysis of its components, the obtained GEO was processed using a gas chromatography-flame ionization detector (Thermo Scientific Focus GC) equipped with an AI 3000 II autosampler, a Stabilwax (Crossbond Carbowax-PEG, Restek) column (60 m length, 0.32 mM internal diameter, 1.0-µm thick film). The extraction rate of GEO (~0.15%) (wt/wt). Citral (purity = 95%, St. Louis, MO, USA) was used to construct the standard curve for the analysis of the citral concentration in GEO. The GEO was composed of citral (20.2%, 175 mg/mL), which contains two isomers, geranial, and neral (Fig. S1). This result was comparable to the previous study (23 – 25).

### Plasma biochemical analyses

The collected blood was centrifuged at 1,000 × *g* for 15 min at 4°C, and the plasma was analyzed by Lezen Reference Lab (Taipei, Taiwan) to determine the levels of total cholesterol, TG, low-density lipoprotein cholesterol, high-density lipoprotein cholesterol, aspartate aminotransferase, and alanine aminotransferase using a Beckman Coulter AU 5800 (Beckman Coulter Inc., USA) (24).

### Liver histopathological analysis

The largest right lobe of the liver was preserved in 10% neutral formalin, followed by histopathological analyses utilizing the formalin-fixed paraffin-embedded technique. The hepatic histological scoring was assessed in the College of Veterinary Medicine Animal Disease Diagnostic Center (National Chung Hsing University, Taichung, Taiwan). The NAFLD activity score was defined by computing scores for steatosis, lobular inflammation, and hepatocyte ballooning (18, 60).

### Hepatic triglyceride and pro-inflammatory cytokine analyses

Hepatic tissue (0.1 g) was homogenized with 1 mL of homogenization buffer (8 mM KH_2_PO_4_, 12 mM K_2_HPO_4_, 1.5% KCl, pH = 7.4) on ice and then centrifuged at 10,000 × *g* for 30 min at 4°C for collection of the supernatant. The hepatic triglyceride content was analyzed using a Triglyceride Colorimetric Assay Kit (Cayman Chemical, USA). The levels of pro-inflammatory cytokines, namely tumor necrosis factor-α, interleukin-1β, and interleukin-6, were analyzed using enzyme-linked immunosorbent assay kits (Invitrogen, USA) (18).

### Western blot

Protein expressions of NLRP3, ASC, caspase-1, TLR4, p-NF-κB/NF-κB, and GAPDH were determined by Western blot as previously described (24). Protein was extracted from 0.1 g liver tissue with 1 mL of chilled lysis buffer (7 M urea, 2 M thiourea, 2% CHAPS, 0.002% bromophenol blue, 60 mM DTT, and a protease and phosphatase inhibitor). The mixture was disrupted with an ultrasonic cell crusher for 5 min on ice thrice and then centrifuged at 17,500 × *g* for 1 h at 4°C. The supernatants were collected, and total protein content was measured using the Bio-Rad Protein Assay Kit following the manufacturer’s instructions (Bio-Rad, USA). All samples were adjusted to the same concentration with sample buffer (62.5 mM Tris-HCl, 10% glycerol, 2% SDS, and 0.01% bromophenol blue), boiled at 95°C for 15 min, and stored at −80℃ until use. Proteins were separated using SDS-polyacrylamide gel electrophoresis (5% and 10% gels) and transferred onto polyvinylidene difluoride membranes (Millipore Corp., Bedford, MA, USA). Unspecific binding was blocked with membrane incubation with 5% bovine serum albumin (BSA) in tris-buffered saline with Tween (TBST) buffer (20 mM Tris-base, 150 mM NaCl, 0.05% Tween 20) for 2 h, followed by three washes with TBST buffer. Membranes were then incubated with the primary antibody (NLRP3, ASC, caspase-1, TLR4, p-NF-κB, NF-κB, and GAPDH) at 4°C for 12–16 h. After incubation, membranes were washed thrice with TBST and incubated with the corresponding horseradish peroxidase-conjugated secondary antibodies (Cell Signaling Technology, USA) at 21–25°C for 2 h. The membrane was then incubated with the enhanced chemiluminescence substrate (ECL, PerkinElmer Life Sciences, Boston, MA, USA) for detection of the enzyme-conjugated antibody, and the signal was captured with an e-BLOT imaging system (Normanda Technology, New Taipei City, Taiwan). Protein expression was quantified by densitometry using the ImageJ software. GAPDH was used as loading control, and the relative phosphorylation was calculated using the ratio of phosphorylated to total protein.

### Fecal microbiota analysis

DNA was extracted from the feces using the QIAmp Power Fecal Pro DNA Kit (QIAGEN, the Netherlands) following the manufacturer’s instructions. The V3-V4 hypervariable region of the 16S rRNA gene was amplified with forward and reverse primers (forward: 5′-TCG TCG GCA GCG TCA GAT GTG TAT AAG AGA CAG CCT ACG GGN GGC WGC AG-3′ and reverse: 5′-GTC TCG TGG GCT CGG AGA TGT GTA TAA GAG ACA GGA CTA CHV GGG TAT CTA ATC C-3′) through PCR in a 25-µL reaction mixture with 5 ng of DNA template, 0.2 µM of forward and reverse primers, and 12.5 µL of 2× Taq Master Mix (KAPA HiFi HotStart ReadyMix, Roche, Switzerland). The PCR cycle conditions were at 95°C for 3 min, followed by 25 cycles at 95°C for 30 s, 55°C for 30 s, 72°C for 30 s, and a final extension step at 72°C for 5 min. The PCR products were cleaned up and quantified before undergoing paired-end sequencing (2 × 300 bp) on the Illumina MiSeq platform. The sequences were processed using the QIIME2 pipeline (https://qiime2.org) with the dada2 plugin to produce ASV sequences against the SILVA database (version 138). The alpha and beta diversity of the fecal microbiota were calculated using the vegan package for R software. Heatmap and correlations were plotted using the heatmap3 and corrplot packages in R, respectively. The gut microbiota functional pathway was predicted using PICRUSt2 (18).

### Lipopolysaccharide quantification

The measurement of hepatic LPS was performed utilizing a cell-based colorimetric assay aimed at identifying biologically active endotoxin. Liver homogenates were introduced into a culture of murine TLR4/NF-κB/SEAP reporter HEK293 cells and incubated for 24 h. The culture medium was then collected and analyzed using the HEK-Blue LPS Detection Kit (InvivoGen, San Diego, CA, USA). The concentration of NF-kB-inducible secreted embryonic alkaline phosphatase was determined using a spectrophotometer at a wavelength of 620 nm, as previously described (24).

### Statistical analysis

Data are represented as the mean ± standard deviation. Two-tailed Student’s *t*-test or one-way ANOVA followed by Tukey’s multiple comparison tests were employed for analyses between different groups. Wilcoxon signed-rank or Kruskal-Wallis tests with Dunn’s multiple comparison tests were used to analyze the NAFLD activity score and the fecal microbiome data set. Spearman’s correlation was conducted to determine the relationship between gut microbiota and obesogenic, metabolic, and NAFLD-related biomarkers. All statistical analyses were performed using GraphPad Prism (version 9.5.1), R (version 4.2.2), or R Studio (version 2022.12.0 + 353).

## Data Availability

The raw 16S rRNA sequencing data are accessible at the NCBI Short Read Archive under the following accession numbers: BioProject: PRJNA966918, BioSample: SAMN34566733, and Sequence Read Archive (SRA): SRR24416510-SRR24416534.

## SUPPLEMENTAL MATERIAL

Supplemental material spectrum.01868-23-s0001.docx Fig. S1 and S2.

ASM does not own the copyrights to Supplemental Material that may be linked to, or accessed through, an article. The authors have granted ASM a non-exclusive, world-wide license to publish the Supplemental Material files. Please contact the corresponding author directly for reuse.
