# Supplementary material for: Healthy diet intervention reverses the progression of NASH through gut microbiota modulation
Source: Microbiol Spectr. 2023 Nov 29;12(1):e01868-23. doi: 10.1128/spectrum.01868-23 (PMC10782987; doi:10.1128/spectrum.01868-23)
Supplement: Supplemental material — Fig. S1 and S2. [file spectrum.01868-23-s0001.docx]

**
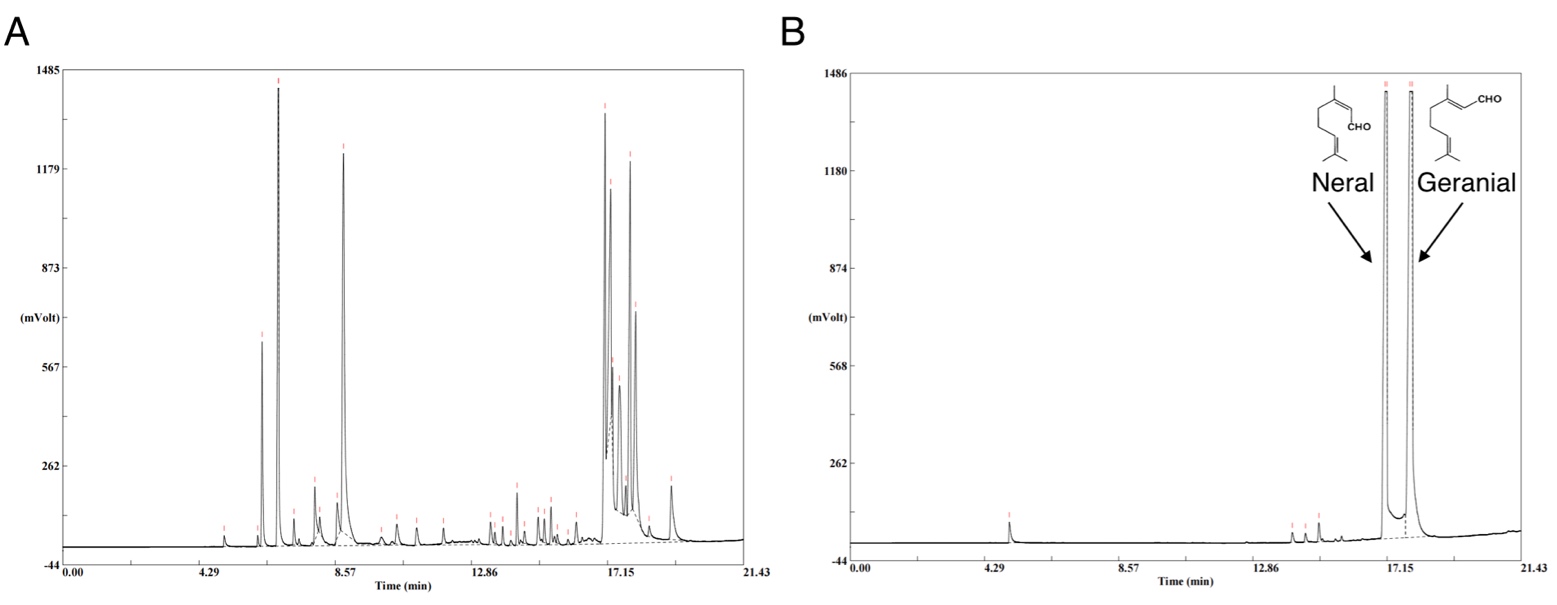
**

**Supplementary Fig. 1. | Gas chromatography of ginger essential oil (GEO) showed citral as its major component. GEO was extracted using steam distillation.** (a) GEO; (b) citral (neral and geranial). Both neral and geranial are geometric isomers of citral.


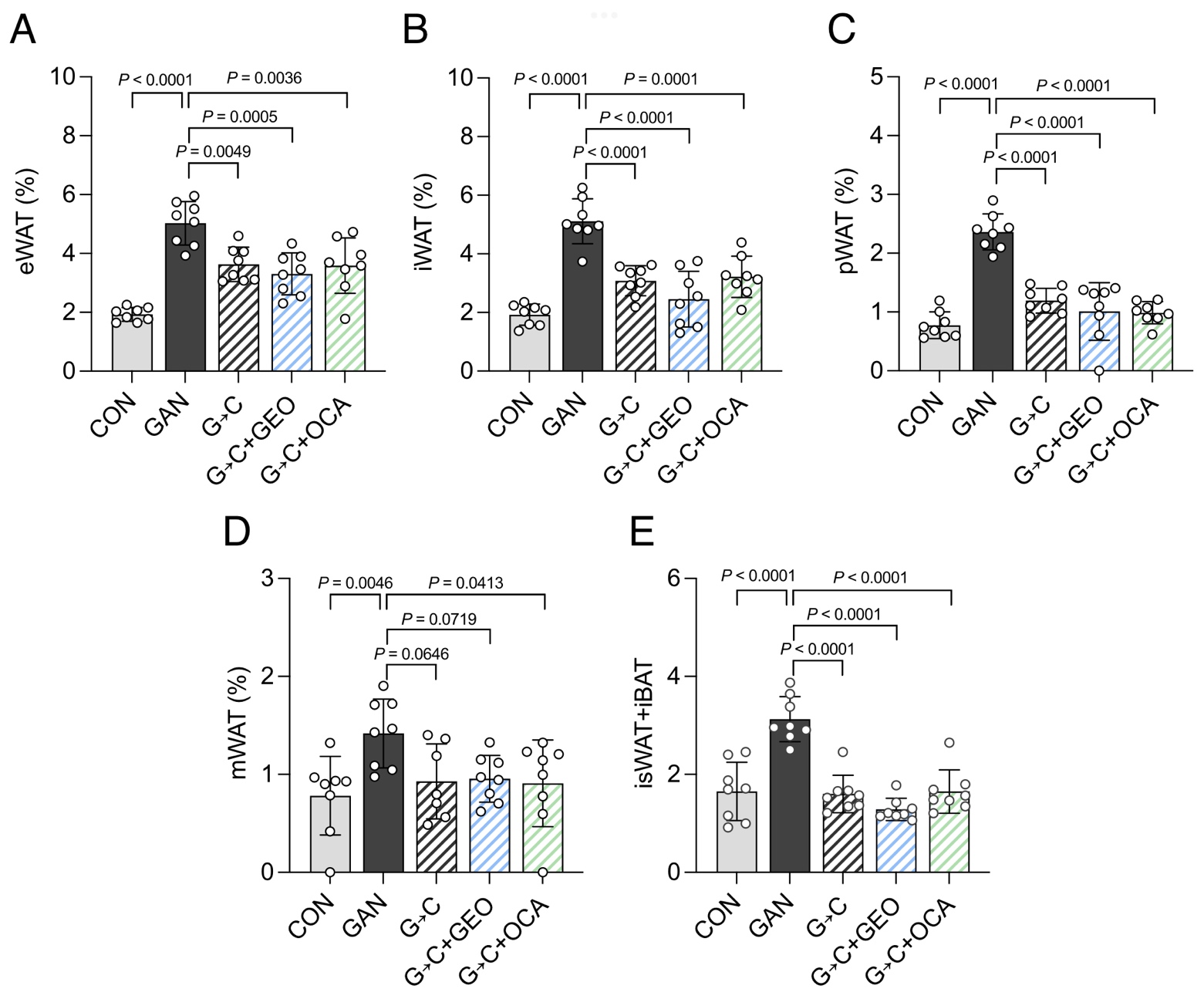


**Supplementary Fig. 2. | Effect of healthy diet intervention, GEO, and OCA on relative fat mass.** (a) Relative epididymal white adipose tissue (WAT) (eWAT), (b) inguinal WAT (iWAT), (c) perirenal WAT (pWAT), (d) mesenteric WAT (mWAT), and (e) inguinal subcutaneous white adipose tissue (isWAT) and interscapular brown adipose tissue (iBAT). Data are represented as the mean ± SD (n = 7–8). Statistical analyses were performed by a two-tailed student's t-test for comparison between CON and GAN groups; and one-way ANOVA with Tukey's range test for comparisons among GAN diet-fed groups. CON, control diet; GAN, Gubra Amylin NASH diet; G→C, change GAN to CON diet; G→C+GEO, diet change with GEO supplementation; and G→C+OCA, diet change with OCA administration.
